# Supplementary figures and images for: Effect of prorenin peptide vaccine on the early phase of diabetic retinopathy in a murine model of type 2 diabetes
Source: PLoS One. 2022 Jan 18;17(1):e0262568. doi: 10.1371/journal.pone.0262568 (PMC8765632; doi:10.1371/journal.pone.0262568)

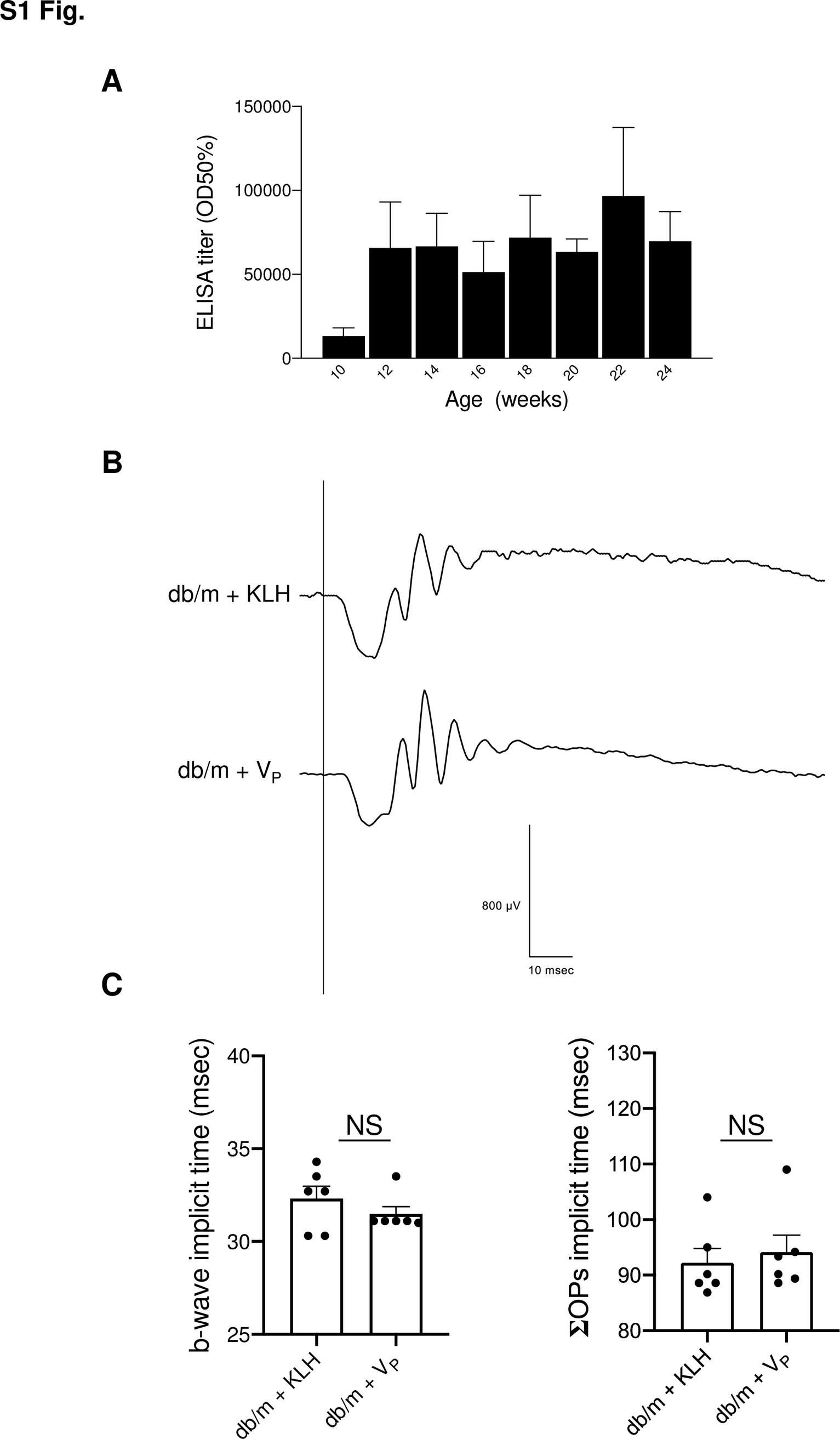

Supplement: S1 Fig — (A) Antibody titer of db/m mice immunized with VP. (B) Electroretinography at 20 weeks of age in db/m mice immunized with keyhole limpet hemocyanin (KLH) control vaccine (db/m + KLH) and VP (db/m + VP). (C) Implicit time of b-wave and total sum of the oscillatory potentials in db/m + KLH and db/m + VP. ΣOPs, total sum of the oscillatory potentials; ELISA, enzyme-linked immunosorbent assay; KLH, keyhole limpet hemocyanin; OD50%, optical density at half-maximal binding; VP, prorenin peptide vaccine. (TIF) [file pone.0262568.s001.tif]

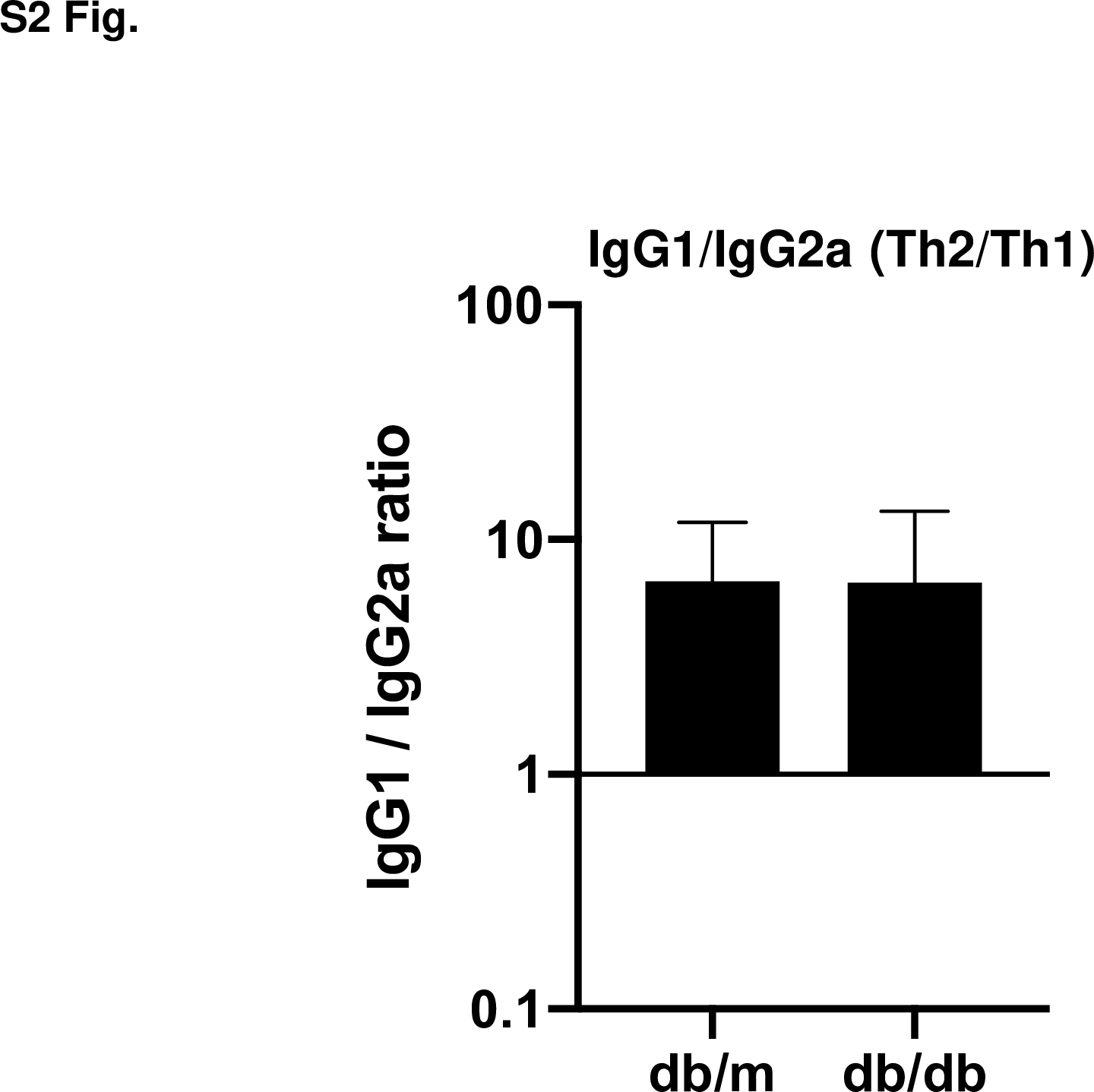

Supplement: S2 Fig — Horseradish peroxidase-conjugated anti-mouse IgG1, IgG2a, IgG2b, and IgG2c (Abcam, Cambridge, MA, USA) were used to determine the IgG subclasses. The IgG1/IgG2a ratio was greater than 1.0, showing that activated T helper 2 cells (Th2) predominated over T helper 1 cells (Th1) in both db/m and db/db mice. (TIF) [file pone.0262568.s002.tif]

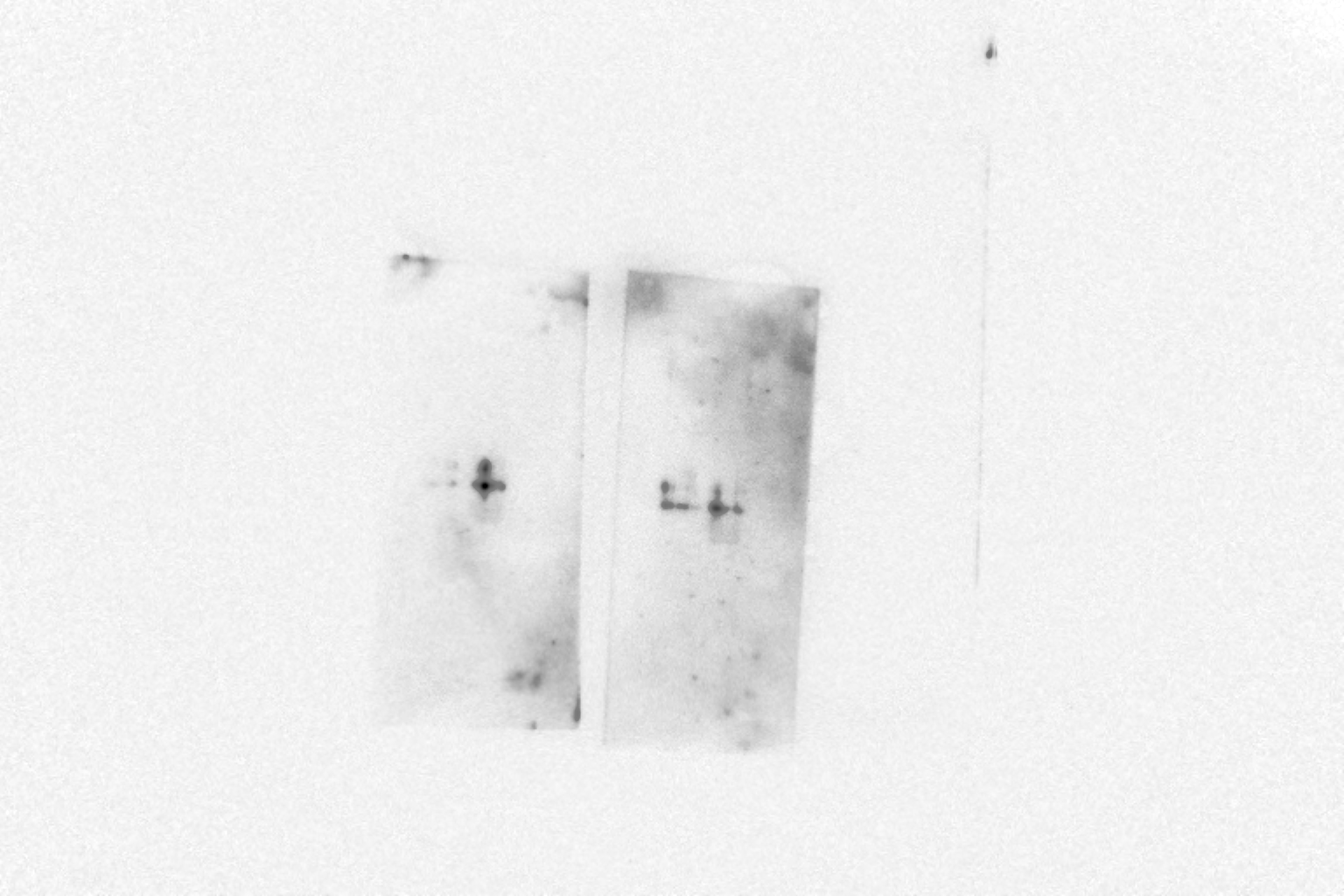

Supplement: S1 Raw image — (TIF) [file pone.0262568.s003.tif]

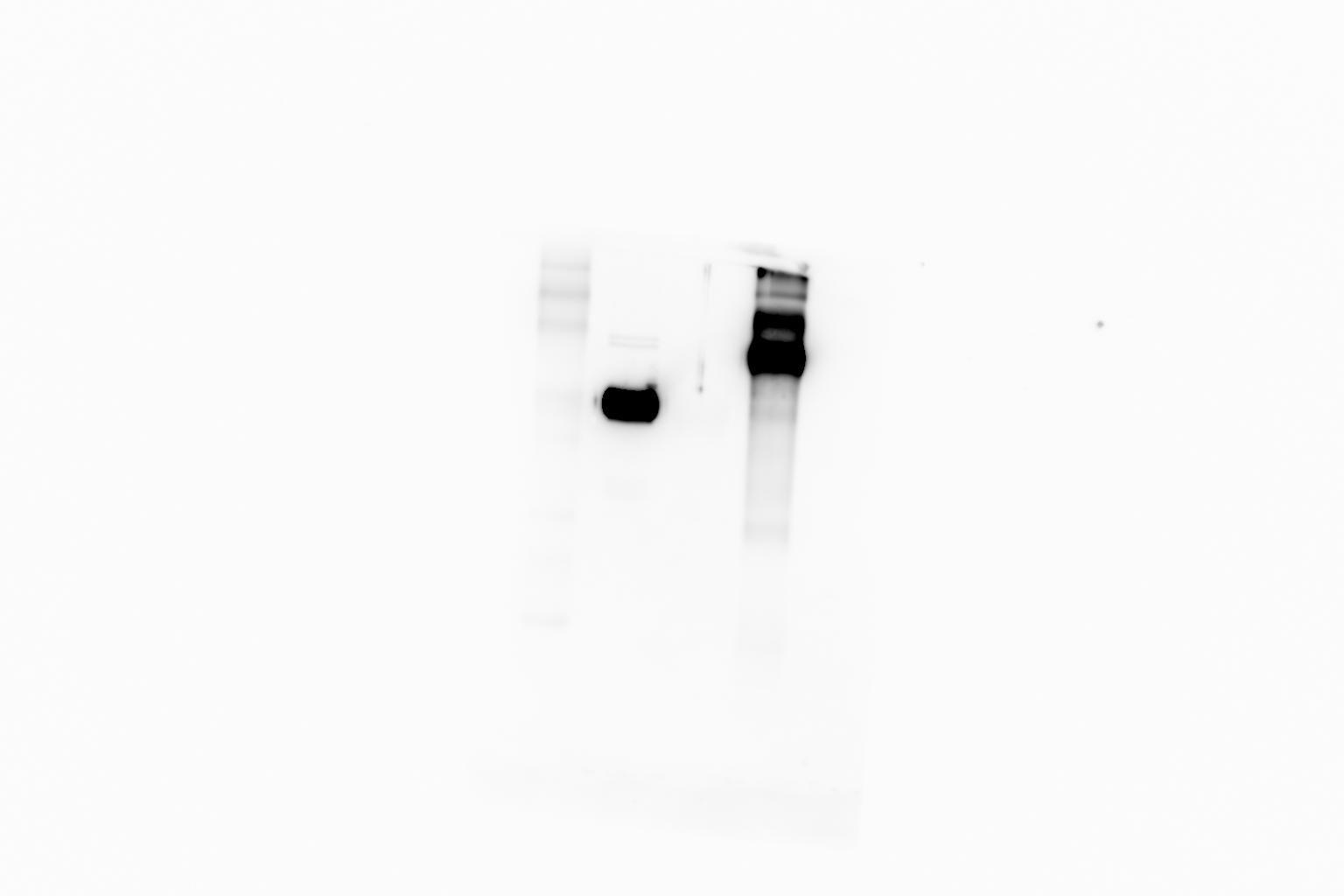

Supplement: S2 Raw image — (TIFF) [file pone.0262568.s004.tiff]

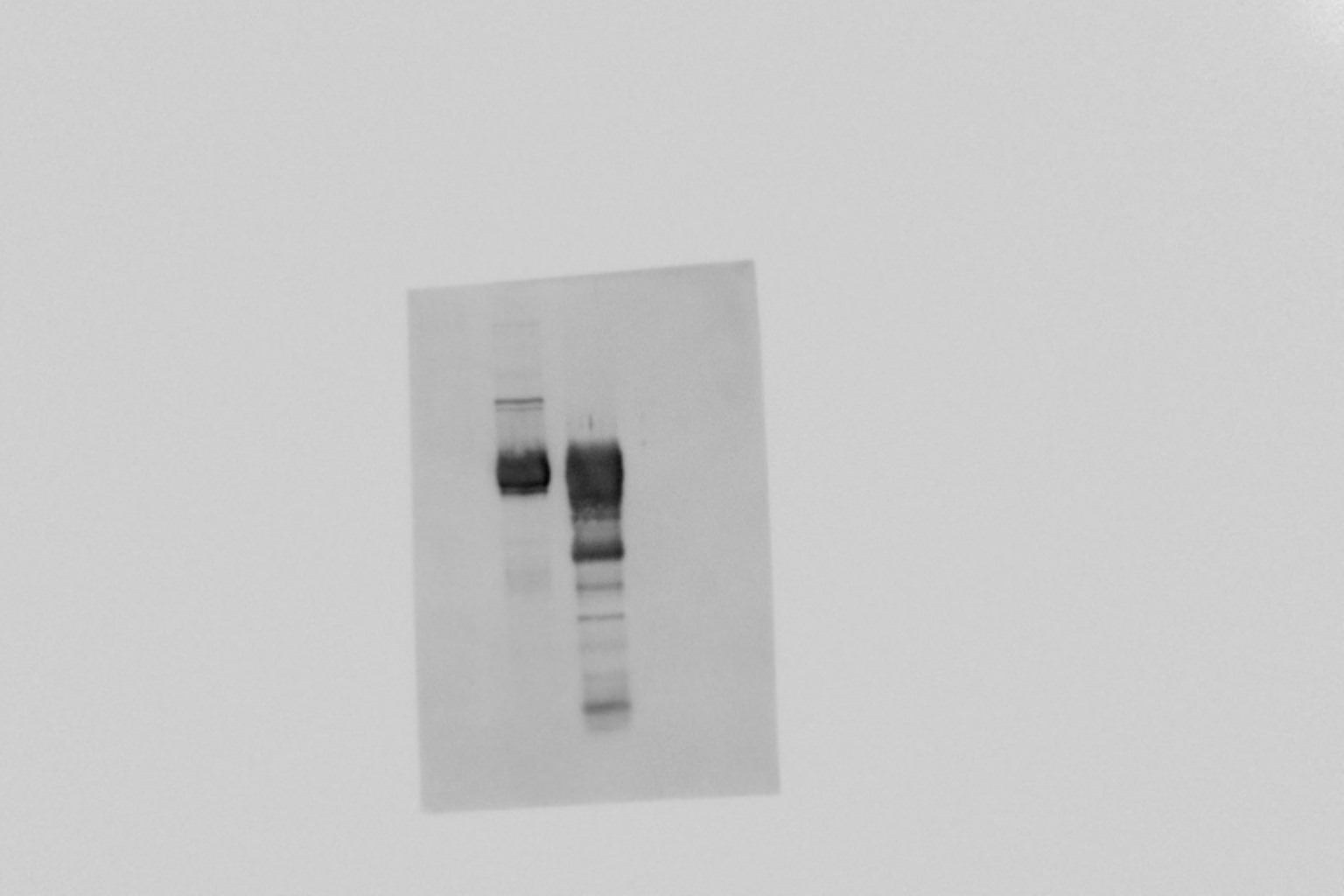

Supplement: S3 Raw image — (TIFF) [file pone.0262568.s005.tiff]
